# The prevalence of dysautonomia in chronic musculoskeletal pain: a systematic review and meta-analysis

**DOI:** 10.1093/rap/rkag034

**Published:** 2026-03-18

**Authors:** Norah A Almutairi, Darren C Greenwood, Manoj Sivan

**Affiliations:** Academic Department of Rehabilitation Medicine, Leeds Institute of Rheumatic and Musculoskeletal Medicine, University of Leeds, Leeds, UK; Physical Therapy and Rehabilitation Department, College of Applied Medical Sciences, Majmaah University, Al Majma’ah, Kingdom of Saudi Arabia; Leeds Institute for Cardiovascular and Metabolic Medicine, School of Medicine, University of Leeds, Leeds, UK; Leeds Institute for Data Analytics, University of Leeds, Leeds, UK; Academic Department of Rehabilitation Medicine, Leeds Institute of Rheumatic and Musculoskeletal Medicine, University of Leeds, Leeds, UK; National Demonstration Centre of Rehabilitation Medicine, Leeds Teaching Hospitals NHS Trust, Leeds, UK

**Keywords:** autonomic dysfunction, orthostatic intolerance, cardiovascular, neuropathic pain, inflammatory, non-inflammatory, arthritis, chronic widespread pain

## Abstract

**Objectives:**

Several chronic musculoskeletal disorders are characterized by pain, fatigue, dizziness and other associated symptoms that may be related to autonomic dysfunction. The aim of this review was to estimate the prevalence of autonomic dysfunction in chronic musculoskeletal pain conditions.

**Methods:**

MEDLINE and Embase were searched through 4 October 2024 for all peer-reviewed studies of dysautonomia in adult musculoskeletal conditions. Risk of bias was assessed using an adapted Newcastle–Ottawa Scale. The prevalence of dysautonomia and relative risk compared with healthy controls were estimated using random effects meta-analysis.

**Results:**

A total of 17 studies (13 fibromyalgia, 3 Ehlers–Danlos syndrome, 1 rheumatoid arthritis) were identified, including 1003 participants with musculoskeletal pain and 417 healthy controls. In people with chronic musculoskeletal pain, the pooled prevalence of dysautonomia was 64% (95% CI 51, 76; *I*^2^ = 93%), more than twice as likely as healthy controls [pooled risk ratio 2.28 (95% CI 1.51, 3.45); *I*^2^ = 24%). Most studies objectively assessed the neurocardiovascular system.

**Conclusion:**

The high prevalence of dysautonomia in patients with chronic musculoskeletal painful conditions illustrates the association between dysautonomia and chronic pain, suggesting regular screening for dysautonomia is warranted for all patients with chronic musculoskeletal pain.

Key messages

- Dysautonomia is not well recognised and often overlooked in patients with painful musculoskeletal conditions.
- Our review suggests 64% of patients with chronic musculoskeletal pain have dysautonomia.
- Chronic musculoskeletal pain patients need to be screened for dysautonomia using subjective and objective assessments to inform a comprehensive management plan.

## Introduction

Chronic musculoskeletal pain (CMP) is defined as continuous or recurring pain that originates directly from bones, joints, muscles or related soft tissues due to an underlying disease process and lasting >3 months [1]. Examples include inflammatory conditions such as RA, osteomyelitis and gout, as well as non-inflammatory or structural conditions like OA and disorders of the tendons or muscles [2].

CMP has been recognised as an important predictor of functional impairment globally [3, 4], requiring assessment and treatment comparable to other chronic conditions [5]. Research in CMP has largely focused on pain-related outcomes, overlooking non-pain symptoms that may significantly influence the overall impact of this condition [6]. Many of these non-pain features may be linked to the autonomic nervous system (ANS), which regulates a wide range of physiological functions and is increasingly recognized as an important contributor to various health conditions, including pain. The ANS may indirectly impact the musculoskeletal system through its role in pain recognition and emotional state regulation [7]. Chronic pain patients have been shown to have a malfunctioning central autonomic network and reduced grey matter, which are also implicated in dysautonomia [8].

Autonomic dysfunction, also known as dysautonomia, is an umbrella term that signifies a disturbance of the ANS. It covers a range of autonomic disorders, including postural orthostatic tachycardia syndrome (POTS), vasovagal syncope, orthostatic hypotension (OH) and undefined conditions with autonomic dysfunction [9]. Dysautonomia may contribute to fatigue, dizziness and other somatic symptoms commonly reported by individuals with CMP [10, 11]. The diagnostic criteria for common autonomic disorders are heart rate increase ≥30 bpm within 10 min of standing without OH for POTS and systolic blood pressure decrease of ≥20 mmHg or a diastolic decrease of ≥10 mmHg within 3 min of standing for OH [12–14]. The commonly used objective tests are head up tilt (HUT), 10-min lean test and adapted autonomic profile test [15].

Dysautonomia is frequently observed in individuals with CMP and may contribute to altered pain modulation [16]. Several CMP conditions are often accompanied by inflammation and oxidative stress, regulated by noradrenaline and acetylcholine, key components of the ANS [17]. However, there is a significant gap in the integration of dysautonomia in the assessment and treatment strategies for CMP, such as arthritis pain [18].

Given the significant interaction between the ANS and the musculoskeletal system, integrating dysautonomia assessment and management into the care plan for individuals with CMP has the potential to improve overall outcomes, including pain reduction. To facilitate translating this into clinical practice, we aimed to estimate the pooled prevalence of dysautonomia among individuals living with a wide range of conditions resulting in CMP.

## Methods

### Protocol and registration

This systematic review is reported in accordance with the Preferred Reporting Items for Systematic Reviews and Meta-Analyses (PRISMA) 2020 guidelines and was prospectively registered in the PROSPERO database (registration number CRD42024594230).

### Search strategy

A literature search was systematically conducted to explore relevant studies available in the MEDLINE and Embase databases (accessed via OVID). The search was restricted to studies in humans only, written in the English language between 1 January 1990 and 4 October 2024. The search terms included synonyms for autonomic dysfunction, dysautonomia, CMP and prevalence, with the full search strategy provided in Supplementary Table S1. The reference lists of the identified publications were manually searched for additional eligible studies.

### Eligibility criteria

Only studies reported in peer-reviewed publications were eligible. All relevant observational studies (i.e. case–control studies, cross-sectional studies, surveys, randomised controlled trials or cohort studies) containing adults ≥18 years of age living with inflammatory or non-inflammatory musculoskeletal conditions where persistent pain is a recognised symptom (e.g. RA or FM) were included. Only studies reporting either standardised patient-reported screening tools or outcome tools or validated objective measures to assess dysautonomia in CMP were included.

We excluded any studies that did not utilise standardised outcome measures or that relied solely on continuous physiological measures (e.g. such as heart rate variability) unless they reported dysautonomia prevalence in their results. All reviews, non-peer-reviewed studies (e.g. conference posters, abstracts or editorials) were excluded. Populations other than those with CMP were also excluded. Where study participants overlapped those from another publication, the more recent publication was selected (Supplementary Table S2).

### Study selection

Two independent reviewers screened titles and abstracts for relevance (N.A.A. and D.C.G.). Disagreements were resolved by consensus or by consulting a third reviewer (M.S.). Full texts were assessed for eligibility based on the predefined criteria. A PRISMA flow diagram (Supplementary Fig. S1) summarises the selection process, including the number of studies screened, excluded (with reasons) and included.

### Data extraction

Data were extracted using a standardized form. Information collected included study characteristics (e.g. author, year, design, sample size), diagnostic methods and the number of confirmed cases of dysautonomia. The extracted data were checked by a second reviewer to ensure accuracy and reliability, with two reviewers participating in the extraction and validation process (N.A.A. and D.C.G.).

### Risk of bias assessment

The quality of included studies was assessed using the Newcastle–Ottawa Scale [19, 20]. The scale evaluated domains for selection, comparability of controls and outcome reporting (Supplementary Table S3), with a maximum total score of 7. A score of 0–3 was interpreted as a high risk of bias, 4–5 as moderate risk and 6–7 as low risk.

### Data synthesis and prevalence estimation

Quantitative data on the prevalence of autonomic dysfunction were pooled using random effects meta-analysis using the Freeman–Tukey transformed proportion to restrict estimates and confidence limits to a range of 0–100% [21]. In cases where different autonomic domains or testing methods were reported separately rather than in combination, the most sensitive testing methods or outcome for detecting dysautonomia was selected for inclusion in the overall prevalence estimate. Meta-analyses were conducted across all studies, within subgroups defined by autonomic outcomes, such as cardiac autonomic dysfunction, and within the underlying musculoskeletal condition, such as FM.

Data for healthy controls were obtained from included studies that reported a control group. The proportion of dysautonomia in people living with CMP was also compared using random effects meta-analysis with the proportion in healthy controls and presented as pooled estimates of the relative risk of dysautonomia.

Heterogeneity was presented as the range of estimates in forest plots and the proportion of total variation attributable to between-study heterogeneity quantified using the *I*^2^ statistic. Small study effects such as publication bias were assessed using funnel plots and the Egger’s test, where at least 10 studies contributed to the meta-analysis.

## Results

### Characteristics of included studies

A total of 1095 records were initially identified through systematic database and citation searching, including two studies identified through manual searching of references [22, 23]. After excluding duplicates, 736 publications were screened for eligibility based on title and abstract and 111 full texts were retrieved for eligibility assessment. One publication was excluded due to potential sample overlap, as indicated by similar recruitment periods and shared authorship [24], with the later publication retained [25]. As illustrated in the PRISMA flow chart (Supplementary Fig. S1), this process resulted in data extraction from 17 studies included in this systematic review.

The key features of the included studies are presented in Table 1. FM was the most studied condition among the CMP conditions, with 13 studies on FM, 3 on Ehlers–Danlos syndrome (EDS) and 1 on RA. A total of 1003 patients with chronic musculoskeletal disease and 417 healthy controls were screened for dysautonomia across the different validated methods and settings. Further information regarding participant demographics and study design characteristics is summarized in Table 2.

**Table 1:** Summary of all included studies.

Study (author, year)	Country	Setting	Medical diagnosis	Sample size, n	Age (years), mean	Female, n	Control group, n	Age (years), mean	Method used	
									Subjective	Objective
De Wandele et al., 2014) [10]	Belgium	Hospital	EDS	80	41	75	0		Autonomic symptom profile	
(De Wandele et al., 2014) [38]	Belgium	Hospital	EDS	39	39	39	35	40		Tilt table test
El-Sawy et al., 2012 [39]	Egypt	Hospital	FM	25	37	23	0			Tilt table test and sympathetic skin response
Furlan et al., 2005 [40]	Italy	Hospital	FM	16	44	15	16	37		Tilt table test
Kulshreshtha et al., 2022 [41]	India	Hospital	FM	42	39	42	0			Modified Ewing’s battery
Lee et al., 2018 [42]	South Korea	Hospital	FM	35	42	35	25	42 (±5 years)		Ewing’s battery
Mucci et al., 2022 [43]	Italy	Community	FM	277	48	248	80	47	Dizziness handicap level	
Naschitz et al., 2006 [25]	Israel	Hospital	FM	70	45	0	50	30		Tilt table test
Oaklander et al., 2013 [44]	Massachusetts	Community	FM	27	47	20	30	45		Autonomic function testing
Seidel et al., 2007 [45]	Germany	Hospital	FM	72	49	72	36	49		Autonomic cardiac dysregulation—ISAX device
Singh et al., 2021 [46]	India	Hospital	FM	30	39	27	30	38		Ewing’s battery
Solano et al., 2009 [47]	Mexico	Hospital	FM	30	47	30	30	39	COMPASS	
Song et al., 2021 [48]	USA	Hospital	EDS	98	38	94	0			Clinical diagnosis of autonomic dysfunction
Stojanovich et al., 2007 [23]	Serbia	Hospital	RA	39	58	33	35	52		Active standing test, Ewing’s battery of tests
Tang, 2004 [49]	USA	Hospital	FM	76	40	72	0			Tilt table test
Vincent et al., 2016 [50]	USA	Community	FM	30	47	30	30	41		COMPASS
Visuri et al., 1992 [51]	Finland	Hospital	FM	17	20	0	20	21	Questionnaire of dystonic symptoms	Active standing test

**Table 2:** Descriptive summary of study populations and methodological characteristics.

Section	Category	n (%)	Participants, n
Study overview	Total studies included	17	1003
	FM	13 (77)	747
	EDS	3 (18)	217
	RA	1 (5)	39
Participant demographics	Age of participants (years), mean	42.29	
	Chronic musculoskeletal participants	1003	
	Control group participants	417	
	Female (chronic musculoskeletal participants)	855 (85)	
	Female (controls)	140 (56)	
Assessment methods	Studies including orthostatic challenge tests	7 (41)	
	Studies including cardiac autonomic function tests	7 (41)	
	Studies including other specific/mixed tests	6 (35)	
	Studies including subjective measures	4 (24)	
Study setting	Hospital-based studies	14 (82)	
	Community-based studies	3 (18)	

### Risk of bias assessment

Three studies (18%) were identified as low risk of bias, 13 studies (76%) as moderate risk and 1 study (6%) as high risk. While most studies met key methodological criteria, some lacked evidence of representative samples or autonomic testing covering a wide range of autonomic function (Supplementary Table S4).

### Overall prevalence

**Figure 1:**
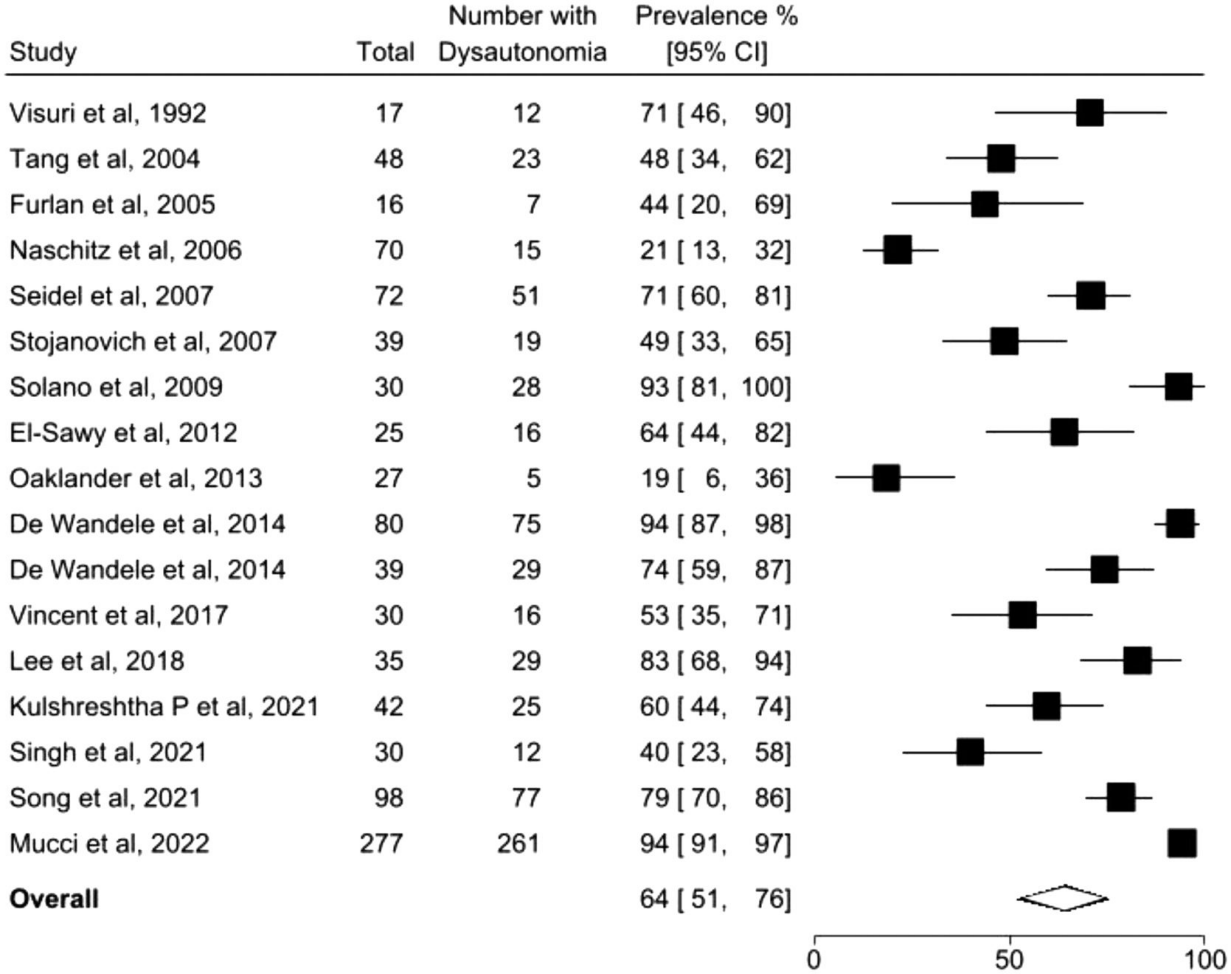
Forest plot shows overall pooled prevalence estimates of dysautonomia in all included musculoskeletal conditions using validated objective and subjective methods

Across all 17 studies in any musculoskeletal condition assessing dysautonomia using any validated autonomic testing method, the estimated pooled prevalence of dysautonomia was 64% (95% CI 51, 76; *I*^2^ = 93%) (Fig. 1). Separate analyses restricted to subjective and objective assessment methods are presented in Supplementary Figs. S2 and S3, respectively.

### Comparison with healthy controls

**Figure 2:**
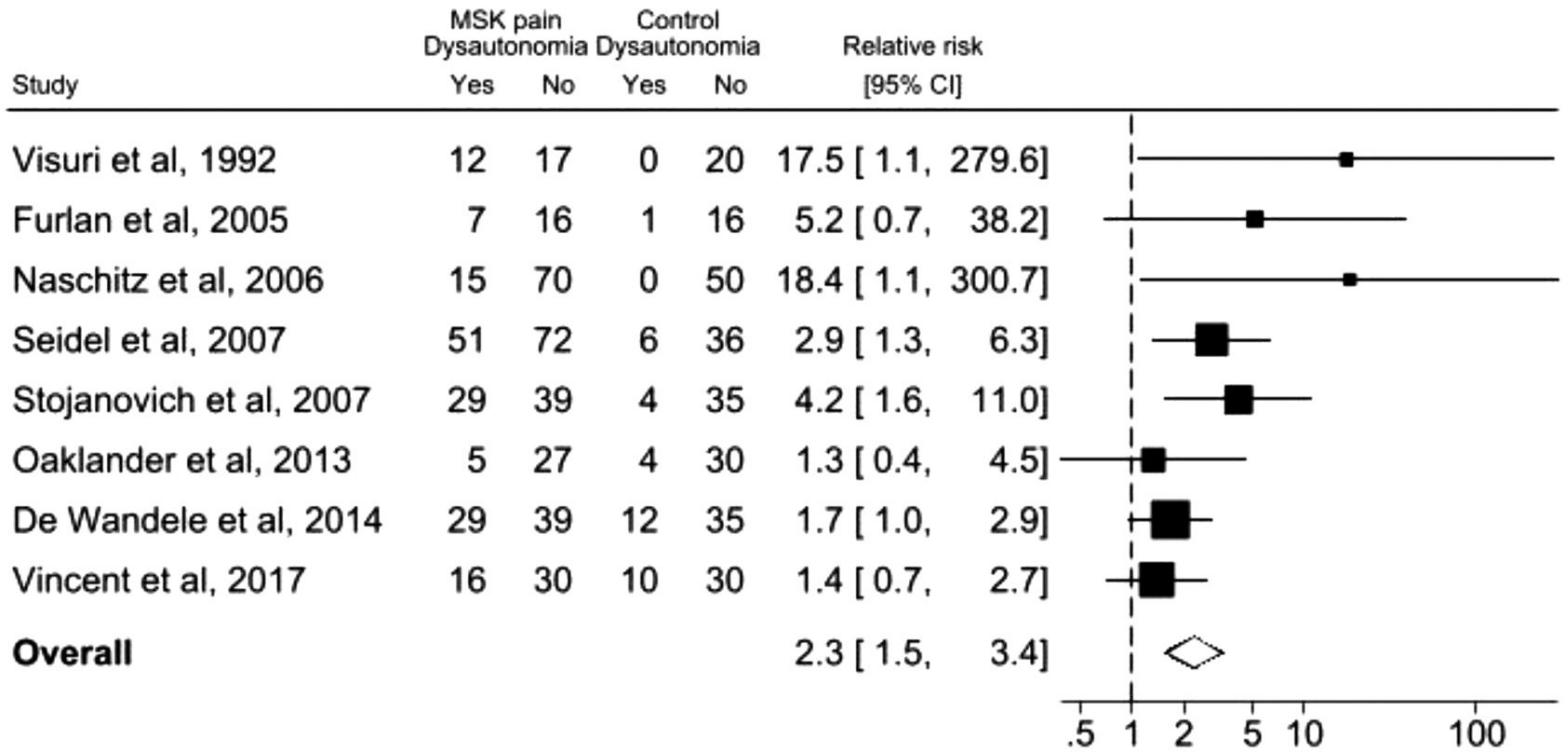
Forest plot of eight studies comparing dysautonomia in musculoskeletal pain patients *vs* healthy controls. Pooled risk ratio 2.3 (95% CI 1.5, 3.4); *I*^2^ = 24.35% (random effects model)

Eight studies (47%) reported sufficient information to allow comparison of dysautonomia in people with CMP and healthy controls, using the same validated autonomic function tests. Individuals with CMP were more than twice as likely to exhibit symptoms of dysautonomia, with a pooled risk ratio of 2.28 (95% CI 1.51, 3.45) and low between-study heterogeneity (*I*^2^ = 24%) (Fig. 2) showing consistency between studies.

#### Subgroups based on autonomic test category and underlying condition

Table 3 presents a summary of the pooled estimates of prevalence of dysautonomia in various chronic musculoskeletal conditions stratified by test category and underlying condition.

**Table 3:** Prevalence of dysautonomia in chronic musculoskeletal conditions, stratified by test category and underlying diagnosis.

Test category	Across various musculoskeletal conditions (FM, RA, EDS)	FM
All measures	*n* = 17; 64% (95% CI 51, 76); *I*^2^ = 93%	*n* = 13; 60% (95% CI 45, 75); *I*^2^ = 93%
All objective	*n* = 13; 56% (95% CI 44, 67); *I*^2^ = 87%	*n* = 11; 52% (95% CI 39, 65); *I*^2^ = 85%
All subjective	*n* = 4; 86% (95% CI 61, 100); *I*^2^ = 95	*n* = 3; 82% (95% CI 44, 100); *I*^2^ = 95%

#### Subgroups based on autonomic test outcomes

Dysautonomia involves a broad range of symptoms; however, the studies included in this review most often reported objective orthostatic challenge tests, such as orthostatic intolerance and/or POTS. Seven studies reported the prevalence of autonomic dysfunction based on orthostatic intolerance identified through orthostatic challenge tests such as the tilt table test or the active standing test, conducted across various musculoskeletal conditions (Supplementary Fig. S4). By this definition, the pooled prevalence of dysautonomia was estimated at 52% (95% CI 37, 67; *I*^2^ = 81%).

In four studies, dysautonomia was assessed using groups of cardiac autonomic objective tests, including cardiac autonomic dysfunction or cardiac autonomic neuropathy. Assessments included standard or modified Ewing’s reflex tests, as well as tools such as the ISAX system to evaluate cardiac autonomic regulation (Supplementary Fig. S5). For the pooled analysis, all positive cases of cardiac dysregulation were considered as dysautonomia. The overall pooled prevalence was 64% (95% CI 46, 81; *I*^2^ = 82%). Additionally, three studies identified POTS using the tilt table test, with a pooled prevalence of 15% (95% CI 0, 44; *I*^2^ = 93%) (Supplementary Fig. S6).

#### Dysautonomia subjective assessment

Four studies reported the prevalence of dysautonomia using subjective measures, including the Autonomic Symptom Profile, Dizziness Handicap Inventory, Composite Autonomic Symptoms Scale (COMPASS) and the Questionnaire of Dystonic Symptoms. The pooled prevalence based on these subjective assessments was higher than that from objective tests, at 86% (95% CI 61, 100; *I*^2^ = 95%). Subgroup analyses for other autonomic assessment methods are provided in Supplementary Fig. S7.

### Subgroups based on underlying conditions

#### FM

**Figure 3:**
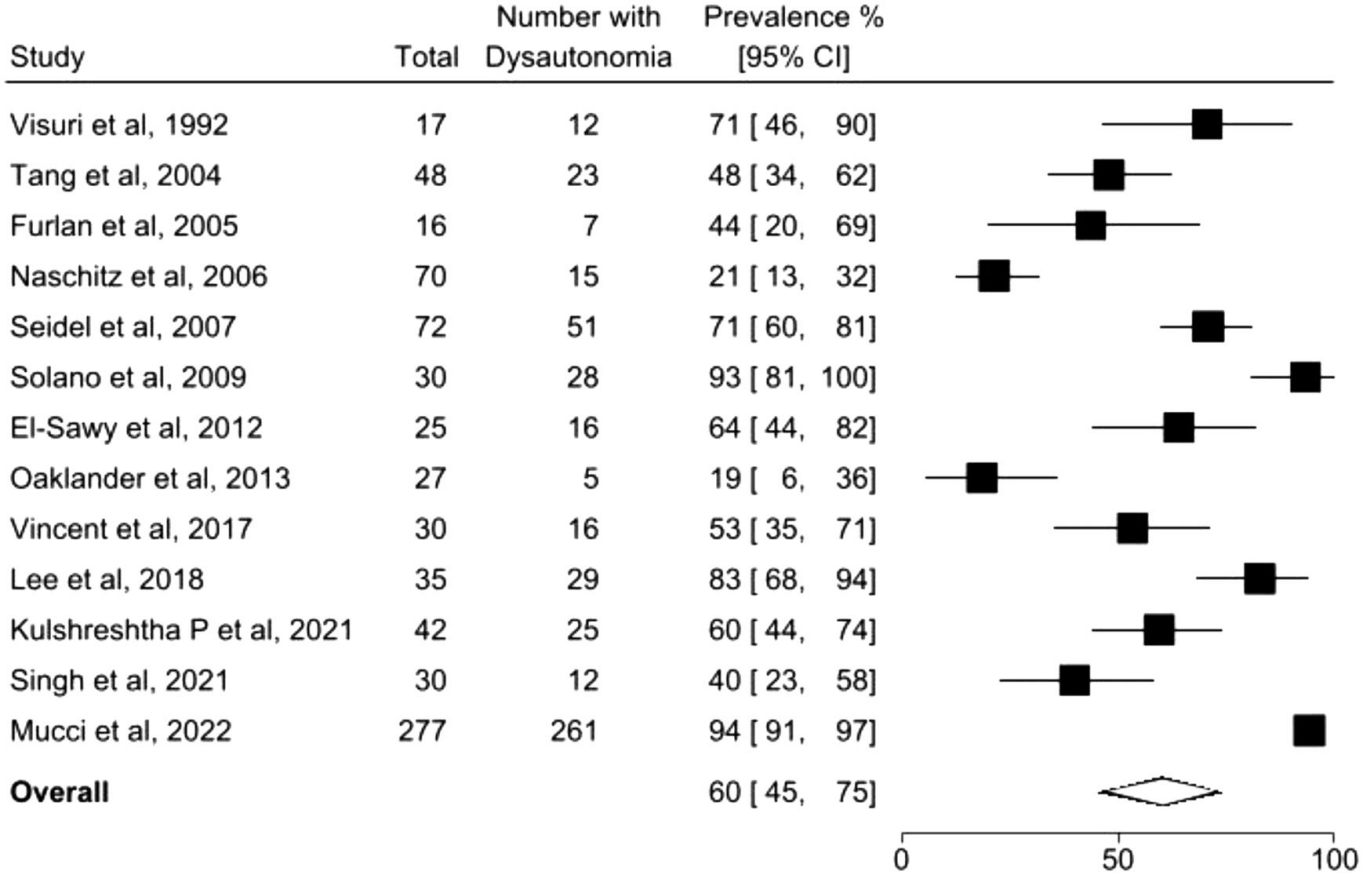
Forest plot shows overall pooled prevalence estimates of dysautonomia in FM using validated objective and subjective methods

FM was the most frequently studied condition among musculoskeletal disorders in this review. A total of 13 studies assessed dysautonomia in FM (Fig. 3), with a pooled prevalence of dysautonomia of 60% (95% CI 45, 75; *I*^2^ = 93%). When classified by assessment method, the prevalence of dysautonomia in FM was estimated at 52% (95% CI 39, 65) when identified through objective testing (Supplementary Fig. S8). In contrast, prevalence reached 82% (95% CI 44, 100) when based on self-report measures (Supplementary Fig. S9). Limiting the analysis to FM studies that examined orthostatic intolerance, the pooled prevalence was 48% (95% CI 30, 66; *I*^2^ = 81%) based on a meta-analysis of five studies (Supplementary Fig. S10).

#### EDS

Three studies examined dysautonomia in EDS, with a pooled prevalence of 84% (95% CI 69, 94), using an objective test in two studies and a subjective method in the other one (Supplementary Fig. S11*)*. We grouped them together to enable prevalence estimation, as only these three studies were available for this condition.

In this review, only one study investigated autonomic dysfunction in an inflammatory condition (RA), so the available evidence was insufficient to calculate the prevalence of dysautonomia separately for non-inflammatory musculoskeletal conditions.

### Small study effects and publication bias

For the meta-analysis of overall prevalence across all studies, the funnel plot and associated Egger’s test (*P* = 0.108) showed no evidence of asymmetry (Supplementary Fig. S12). Equivalent funnel plots based on objective and subjective assessment methods separately are presented in Supplementary Figs. S13 and S14. There were insufficient studies reporting dysautonomia based on orthostatic intolerance or other cardiovascular outcomes to evaluate potential small study effects such as publication bias for these outcomes (Supplementary Figs. S15 and S16). There was no evidence of funnel plot asymmetry for people living with FM (Supplementary Figs. S17–S19) (*P* = 0.342), but there were insufficient studies to evaluate potential asymmetry for comparison with healthy controls or for other subgroups.

## Discussion

This review found that neurocardiovascular-specific markers were the most examined features of dysautonomia within chronic musculoskeletal populations. These markers include both objective indicators and subjective symptoms related to cardiovascular autonomic function. Neurocardiovascular-related signs (e.g. orthostatic intolerance, orthostatic hypotension, POTS) are measurable, standardized and provide reliable, reproducible measures of autonomic function, making prevalence estimates more consistent and valid across studies.

Our meta-analysis estimated the pooled prevalence of dysautonomia to be >50% of individuals living with CMP, and more prevalent than in healthy controls. These findings are broadly consistent with previous small clinical studies in specific patient groups. For instance, Galosi *et al*. [26] revealed that ≈50% of FM patients showed evidence of small fibre neuropathy, an indirect indicator of autonomic dysfunction. Similarly, dysautonomia was identified in 65% of females and 44% of males with hypermobile EDS, further supporting the high prevalence of autonomic disturbances across related chronic musculoskeletal conditions [27]. A recent study [28] reported that 90% of patients with hypermobile EDS (with pain) had autonomic failure on testing, further reinforcing the finding of our review.

While many previous reviews have discussed the qualitative aspects of dysautonomia in FM and musculoskeletal pain, they have generally lacked quantitative synthesis or prevalence estimates [29, 30]. Most existing studies have involved small sample sizes or focused primarily on continuous physiological measures such as heart rate variability without adequately quantifying how many of the participants meet the criteria for dysautonomia.

Inadequate identification of dysautonomia has prevented it from being recognised as a standard factor in clinical management. Our review fills this gap by contributing valuable epidemiological data to the existing literature and offering a clearer picture of the clinical burden of dysautonomia in the CMP population, which ultimately raises clinicians’ awareness to consider this condition when assessing CMP patients and manage its impact more effectively. Our review findings provide further evidence to support a recent article [31] calling for routine dysautonomia screening in systemic conditions.

Although our estimate is slightly higher, it may be explained and supported by physiological evidence from Bruehl *et al.* [32], one of the largest population-based investigations to assess autonomic function in chronic pain. More than 1100 participants with chronic pain were compared with 5600 pain-free controls using heart rate variability (HRV) and baroreflex sensitivity (BRS) derived from continuous cardiovascular recordings. The results showed significantly reduced HRV and BRS in the chronic pain group, indicating dysautonomia. While this study did not quantify prevalence directly, its rigorous methodology and large sample size provide a substantial contribution to the physiological evidence base supporting the prevalence estimates reported in our meta-analysis. Together, these findings underscore the importance of routine autonomic assessment in CMP populations.

Given the ANS’s broad systemic effects, dysautonomia symptoms can be diverse and encompass symptoms from all systems (e.g. cardiac, somatic, gastrointestinal). This can introduce significant variation in estimated prevalence, depending on the methods used. While we have explored a number of predefined subgroups based on the aspects of dysautonomia assessed, and based on the type of musculoskeletal condition, substantial between-study heterogeneity remained. However, when making within-study comparisons with healthy controls, most heterogeneity was eliminated and indicated that people with CMP were more than twice as likely to have dysautonomia.

It is worth noting that four studies in this review incorporated subjective methods to report dysautonomia prevalence within CMP and only one of them did so in combination with objective assessments, while the remaining studies either relied solely on one objective measure or used combinations of objective tests. This underscores the limited incorporation of patient-reported outcome measures (PROMs) in the existing literature and highlights the need for future studies to include both PROMs and objective tests when investigating dysautonomia in CMP.

Across the 17 studies included in this review, only one investigated autonomic dysfunction in an inflammatory condition (RA), whereas the remaining studies focused on non-inflammatory conditions. Given the limited evidence on inflammatory conditions, a direct comparison of prevalence between inflammatory and non-inflammatory groups was not feasible and the current evidence base is insufficient to draw firm conclusions. Future studies are warranted to address this gap.

Dysautonomia has been observed in other chronic painful conditions such as chronic fatigue syndrome and long COVID, indicating potential shared underlying mechanisms [33–35]. Early screening for dysautonomia is essential, as diagnosis currently takes an average of 7.7 years [36]. Validated PROMs including COMPASS-31 and SPIDER followed by an in-clinic 10-min active standing/lean test can facilitate early objective detection of dysautonomia in CMP as a cost-effective screening measure. Patients who screen positive can be referred to more advanced autonomic diagnostic testing such as HUT or tilt table tests.

Management of dysautonomia may begin with non-pharmacological approaches, including lifestyle modifications such as increased fluid and salt intake, use of compression garments, avoidance of prolonged standing and high temperatures and calf exercise programs. Pharmacological options including beta blockers/ivabradine and midodrine/fludrocortisone may be considered later if the patient’s response is insufficient [37].

### Limitations

Two studies by De Wandele *et al*. [10, 38] were conducted at the same research centre with a potential possibility of overlapping patient cohorts. However, these studies employed different assessment methods (objective autonomic testing *vs* subjective questionnaire), indicating they were separate groups. Although potential overlap in the overall pooled estimate cannot be excluded, sensitivity analyses carried out excluding each study in turn had no influence on conclusions.

This meta-analysis is primarily based on studies of FM, with limited data on other musculoskeletal conditions such as EDS and RA. As a result, the generalisability of the findings to broader musculoskeletal populations may be limited. We did not include hypermobility spectrum disorder or small fibre neuropathy in the conditions, which restricted the search. While the pooled prevalence indicates a substantial burden, the narrow scope of conditions and differences in autonomic testing methods may have introduced heterogeneity. Research that standardises assessment practices for people with CMP and research in rarer or less-easily investigated conditions may add further insight.

## Conclusion

Dysautonomia can lead to several health challenges, including but not limited to orthostatic intolerance, cardiac dysfunction, pain and fatigue. Two-thirds of CMP patients had some degree of dysautonomia when assessed using subjective and objective tests. This highlights the importance of clinicians screening for dysautonomia and managing it appropriately within a comprehensive management plan for CMP.

## Supplementary Material

rkag034_Supplementary_Data

## Data Availability

Data are available upon reasonable request to the corresponding author.
