# Supplementary material for: The prevalence of dysautonomia in chronic musculoskeletal pain: a systematic review and meta-analysis
Source: Rheumatol Adv Pract. 2026 Mar 18;10(2):rkag034. doi: 10.1093/rap/rkag034 (PMC13070644; doi:10.1093/rap/rkag034)

**Supplementary Materials**

Contents

[Supplementary Tables 3](#_Toc223941768)

[Supplementary Table 1. Search strategy 3](#_Toc223941769)

[Supplementary Table 2. List of studies excluded at full text screening stage with brief reason 3](#_Toc223941770)

[Supplementary Table 3. Adapted Newcastle - Ottawa Quality Assessment Scale 11](#_Toc223941771)

[Supplementary Table 4. Risk of Bias Assessment 13](#_Toc223941772)

[Supplementary Figure 1. PRISMA flowchart demonstrates the article screening process. 18](#_Toc223941773)

[Forst plots 19](#_Toc223941774)

[Supplementary Figure 2. *Forest plot shows overall pooled prevalence estimates of Dysautonomia across various musculoskeletal conditions using ONLY subjective methods* 19](#_Toc223941775)

[Supplementary Figure 3. Forest plot shows overall pooled prevalence estimates of Dysautonomia across various musculoskeletal conditions using ONLY objective methods 19](#_Toc223941776)

[Supplementary Figure 4. Forest plot shows overall pooled prevalence estimates of Dysautonomia in all included musculoskeletal conditions using orthostatic intolerance domain. 20](#_Toc223941777)

[Supplementary Figure 5***.*** Forest plot shows overall pooled prevalence estimates of Dysautonomia in all included musculoskeletal conditions using groups of autonomic tests, and only report a single cardiac-related outcome (cardiac autonomic dysfunction (CAD) or cardiac autonomic neuropathy (CAN) 20](#_Toc223941778)

[Supplementary Figure 6***.*** Forest plot shows overall pooled prevalence estimates of POTS in all included musculoskeletal conditions using Tilt table test 20](#_Toc223941779)

[Supplementary Figure 7. Forest plot shows overall pooled prevalence estimates of Dysautonomia in all included musculoskeletal conditions using mixed clinical and subclinical testing approaches 21](#_Toc223941780)

[Supplementary Figure 8*. Forest plot shows the overall pooled prevalence of dysautonomia in fibromyalgia, based on validated objective assessment methods.* 21](#_Toc223941781)

[*Supplementary Figure 9. Forest plot shows the overall pooled prevalence of dysautonomia in fibromyalgia, using self-report methods*. 22](#_Toc223941782)

[*Supplementary Figure* 10. *Forest plot shows overall pooled prevalence estimates of Dysautonomia in fibromyalgia using orthostatic intolerance domain.* 22](#_Toc223941783)

[*Supplementary Figure 11. Forest plot shows the overall pooled prevalence of dysautonomia in Ehlers-Danlos syndrome, based on validated objective and subjective assessment methods.* 22](#_Toc223941784)

[Funnel plots 23](#_Toc223941785)

[Supplementary Figure 12. *Funnel plot of dysautonomia prevalence estimates across all included studies and assessment methods.* 23](#_Toc223941786)

[Supplementary Figure 13. Funnel plot of dysautonomia prevalence estimates across all included studies using objective assessment methods 23](#_Toc223941787)

[Supplementary Figure14. *Funnel plot of dysautonomia prevalence estimates across all included studies using subjective assessment methods* 24](#_Toc223941788)

[Supplementary Figure 15*. Funnel plot of dysautonomia prevalence estimates across all included studies based on orthostatic domain assessment* 24](#_Toc223941789)

[Supplementary Figure 16. *Funnel plot of dysautonomia prevalence estimates across all included studies based on mixed clinical and subclinical testing approaches.* 24](#_Toc223941790)

[Supplementary Figure 17. Funnel plot of dysautonomia prevalence estimates in fibromyalgia using various assessment methods. 25](#_Toc223941791)

[Supplementary Figure 18***.*** Funnel plot of dysautonomia prevalence estimates in fibromyalgia using objective assessment methods 25](#_Toc223941792)

[Supplementary Figure 19. Funnel plot of dysautonomia prevalence estimates in fibromyalgia based on orthostatic domain assessment 26](#_Toc223941793)

# Supplementary Tables

## Supplementary Table 1. Search strategy

1. (Autonomic Dysfunction* OR Dysautonomia* OR Autonomic Dysregulation* OR Parasympathetic Activity* OR Sympathetic Activity*).tw.

2. (CASS* OR COMPASS* OR Tilt Table* OR Stabilometry* OR COMPASS 31* OR Autonomic Function Testing* OR Ewing's Battery of Tests* OR Valsalva Maneuver* OR Orthostatic Hypotension* OR Heart Rate Variability* OR Postural Tachycardia* OR Orthostatic Intolerance*).tw.

3. (Musculoskeletal* OR MSK* OR Musculoskeletal System* OR Osteoarthritis* OR Rheumatoid Arthritis* OR Rheumatism* OR LBP* OR Chronic Musculoskeletal Condition* OR Fibromyalgia*).tw.

4. 1 OR 2

5. 3 AND 4

6. exp Animals/ NOT (exp Humans/)

7. 5 NOT 6

8. 7 NOT ((exp child/ OR exp adolescent/) NOT exp adult/)

9. Limit 8 to English Language

10. Limit 9 to yr="1990 -Current"

## Supplementary Table 2. List of studies excluded at full text screening stage with brief reason

**Review**

1. Kocyigit, B.F., Akyol, A., 2023. Coexistence of fibromyalgia syndrome and inflammatory rheumatic diseases, and autonomic cardiovascular system involvement in fibromyalgia syndrome. Clin Rheumatol 42, 645–652. https://doi.org/10.1007/s10067-022-06385-8
2. Koopman, F.A., Stoof, S.P., Straub, R.H., van Maanen, M.A., Vervoordeldonk, M.J., Tak, P.P., 2011. Restoring the Balance of the Autonomic Nervous System as an Innovative Approach to the Treatment of Rheumatoid Arthritis. Mol Med 17, 937–948. https://doi.org/10.2119/molmed.2011.00065
3. Malkova A.M., Shoenfeld Y., 2023. Autoimmune autonomic nervous system imbalance and conditions: Chronic fatigue syndrome, fibromyalgia, silicone breast implants, COVID and post-COVID syndrome, sick building syndrome, post-orthostatic tachycardia syndrome, autoimmune diseases and autoimmune/inflammatory syndrome induced by adjuvants. Autoimmunity Reviews 22, 103230-undefined. https://doi.org/10.1016/j.autrev.2022.103230
4. Shim, H., Rose, J., Halle, S., Shekane, P., 2019. Complex regional pain syndrome: a narrative review for the practising clinician. British Journal of Anaesthesia 123, e424–e433. https://doi.org/10.1016/j.bja.2019.03.030
5. Yeater T.D., Cruz C.J., Cruz-Almeida Y., Allen K.D., 2022. Autonomic Nervous System Dysregulation and Osteoarthritis Pain: Mechanisms, Measurement, and Future Outlook. Current Rheumatology Reports 24, 175–183. <https://doi.org/10.1007/s11926-022-01071-9>
6. Buskila, D., 2000. Fibromyalgia, chronic fatigue syndrome, and myofascial pain syndrome. Current Opinion in Rheumatology 12, 113.

**Editorial**

1. Eisinger J., 2007. Dysautonomia, fibromyalgia and reflex dystrophy. Arthritis Research and Therapy 9. https://doi.org/10.1186/ar2212
2. Martinez-Lavin M., Hermosillo A.G., 2005. Dysautonomia in Gulf War syndrome and in fibromyalgia [3]. American Journal of Medicine 118. https://doi.org/10.1016/j.amjmed.2004.11.023
3. Onrat E., Akci O., 2017. Comparison of heart rate variability and classic autonomic testing for detection of cardiac autonomic dysfunction in patients with fibromyalgia. International Journal of Rheumatic Diseases 20, 2140-undefined. https://doi.org/10.1111/1756-185X.13058

**Target population**

1. Bar-On, E., Floman, Y., Sagiv, S., Katz, K., Pollak, R.D., Maayan, C., 2000. Orthopaedic Manifestations of Familial Dysautonomia : A Review of One Hundred and Thirty-six Patients*. JBJS 82, 1563.
2. Chelimsky, G., Kovacic, K., Nugent, M., Mueller, A., Simpson, P., Chelimsky, T.C., 2015. Comorbid Conditions Do Not Differ in Children and Young Adults with Functional Disorders with or without Postural Tachycardia Syndrome. The Journal of Pediatrics 167, 120–124. https://doi.org/10.1016/j.jpeds.2015.03.039
3. Christopoulos E.M., Reijnierse E.M., Lange P.W., Meskers C.G.M., Maier A.B., 2021. Orthostatic Hypotension and Orthostatic Intolerance Symptoms in Geriatric Rehabilitation Inpatients, RESORT. Journal of the American Medical Directors Association 22, 2468-2477.e2. https://doi.org/10.1016/j.jamda.2021.08.014
4. Kovacic K., Chelimsky T.C., Sood M.R., Simpson P., Nugent M., Chelimsky G., 2014. Joint hypermobility: A common association with complex functional gastrointestinal disorders. Journal of Pediatrics 165, 973–978. https://doi.org/10.1016/j.jpeds.2014.07.021
5. Liporaci R.F., Saad M.C., Crescencio J.C., Marques F., Bevilaqua-Grossi D., Gallo-Junior L., 2018. Cardiac and musculoskeletal responses to the effects of passive and active tilt test in healthy subjects. Arquivos Brasileiros de Cardiologia 110, 74–83. https://doi.org/10.5935/abc.20180003
6. Mehta R.K., 2015. Impacts of obesity and stress on neuromuscular fatigue development and associated heart rate variability. International Journal of Obesity 39. https://doi.org/10.1038/ijo.2014.127
7. Miwa K., Inoue Y., 2023. Repetitive transcranial magnetic stimulation ameliorates symptoms in patients with myalgic encephalomyelitis (chronic fatigue syndrome). IBRO Neuroscience Reports 15. https://doi.org/10.1016/j.ibneur.2023.10.008
8. Oaklander, A.L., Klein, M.M., 2013. Evidence of Small-Fiber Polyneuropathy in Unexplained, Juvenile-Onset, Widespread Pain Syndromes. Pediatrics 131, e1091–e1100. https://doi.org/10.1542/peds.2012-2597
9. Rayhan R.U., Baraniuk J.N., 2021. Submaximal Exercise Provokes Increased Activation of the Anterior Default Mode Network During the Resting State as a Biomarker of Postexertional Malaise in Myalgic Encephalomyelitis/Chronic Fatigue Syndrome. Frontiers in Neuroscience 15, 748426-undefined. https://doi.org/10.3389/fnins.2021.748426
10. Rodrigues P.T.V., Correa L.A., Reis F.J.J., Meziat-Filho N.A., Silva B.M., Nogueira L.A.C., 2021. One Session of Spinal Manipulation Improves the Cardiac Autonomic Control in Patients with Musculoskeletal Pain: A Randomized Placebo-Controlled Trial. Spine 46, 915–922. https://doi.org/10.1097/BRS.0000000000003962
11. Santos A., De Melo V., Luiz Santos A., Tavares L., Araujo F., Santana-Filho V., De Santana J., 2021. Effect of transcutaneous electrical nerve stimulation on autonomic cardiovascular nervous system of women with fibromyalgia: A randomized controlled trial. Biomedical and Biopharmaceutical Research 18. https://doi.org/10.19277/bbr.18.2.265

Not validated outcome

1. Chan, C., Krahe, A., Lee, Y.T., Nicholson, L.L., 2019. Prevalence and frequency of self-perceived systemic features in people with joint hypermobility syndrome/Ehlers-Danlos syndrome hypermobility type. Clin Rheumatol 38, 503–511. https://doi.org/10.1007/s10067-018-4296-7
2. Ulas U.H., Unlu E., Hamamcioglu K., Odabasi Z., Cakci A., Vural O., 2006. Dysautonomia in fibromyalgia syndrome: Sympathetic skin responses and RR Interval analysis. Rheumatology International 26, 383–387. https://doi.org/10.1007/s00296-005-0007-1

Conference abstract and posters

1. Cepoi-Bulgac D., 2018. Assessment of autonomic dysfunction in fibromyalgia patients using compass 31. Osteoporosis International 29, S500-undefined. <https://doi.org/10.1007/s00198-018-4465-1>
2. De Wandele I., Rombaut L., Malfait F., De Paepe A., Calders P., 2011. Presence and impact of autonomic symptoms in patients with the Ehlers-Danlos syndrome. Physiotherapy (United Kingdom) 97, eS272-undefined. https://doi.org/10.1016/j.physio.2011.04.002
3. Eccles J., Amato M., Themelis K., Quadt L., Critchley H., Harrison N., Davies K., 2023. INFLAMMATION-INDUCED PAIN AND FATIGUE IN FIBROMYALGIA AND ME/CFS AND ROLE OF VARIANT CONNECTIVE TISSUE. Annals of the Rheumatic Diseases 82, 129-undefined. https://doi.org/10.1136/annrheumdis-2023-eular.5642
4. Esber A., Malysheva O., Baum P., Baerwald C., Del Rey A., Kolbe I., 2011. Impact of stress on autonomic modulation in patients with rheumatoid arthritis. NeuroImmunoModulation 18. https://doi.org/10.1159/000331256
5. Falco P., Galosi E., Esposito N., Di Pietro G., De Stefano G., Leone C., Di Stefano G., Truini A., 2022. AUTONOMIC DYSFUNCTION IN FIBROMYALGIA: NEW INSIGHTS FROM THE SKIN. Neurological Sciences 43. https://doi.org/10.1007/s10072-022-06531-9
6. Karatas D., Erdik N., Yavuzbilge G., Acikgoz S.B., Turk S.M., Arslanturk Guneysu C., Ozturk Z., Gonullu E., 2023. IS AUTONOMIC DYSFUNCTION MORE COMMON IN PATIENTS WITH PRIMARY SJOGREN’S SYNDROME WITH FIBROMYALGIA THAN IN PATIENTS WITHOUT FIBROMYALGIA? Annals of the Rheumatic Diseases 82, 1553-undefined. https://doi.org/10.1136/annrheumdis-2023-eular.6081
7. Ko K.M., Moon S.-J., 2018. Association of sudoscan values with disease duration in female patients with fibromyalgia. Arthritis and Rheumatology 70. https://doi.org/10.1002/art.40700
8. Lautenschlager G., Thieme K., Malinowski R., Meller T., Kaps M., Kramer H., 2015. Sympathetic activity in patients with fibromyalgia-A microneurographic study. Clinical Neurophysiology 126. https://doi.org/10.1016/j.clinph.2015.04.166
9. Martinez-Lavin M., Lerma C., Martinez-Martinez L.A., Infante O., Vargas A., 2013. Autonomic nervous system “decomplexification” in fibromyalgia a proof of concept study looking at the fractality of heart rhythms. Arthritis and Rheumatism 65. https://doi.org/10.1002/art.38216
10. Martinez-Martinez A., Gonzalez C.L., Del Carmen Navarro Gonzalez M., Vazquez O.I., Trejo F.I.L., Cano E.A., Aguayo A.S., Gomez D.P., Villalpando M.I.B., Silveira L., Ortiz V.H., Guerrero A.V., Martinez-Lavin M., 2019. Correlation between heart rate variability parameters and circulating neuropeptides in fibromyalgia laura. Arthritis and Rheumatology 71, 349–350. <https://doi.org/10.1002/art.41108>
11. Martinez-Martinez L.-A., Perez M.C., Reyes-Loyola P., Martinez-Lavin M., 2015. Fibromyalgia dysautonomia and distress. Correlation between the newly developed composite autonomic symptoms (COMPASS-31) questionnaire and the fibromyalgia polysymptomatic distress scale. Arthritis and Rheumatology 67. <https://doi.org/10.1002/art.39448>
12. Pickering G., Macian N., Achard A., Cardot J.-M., Pereira B., 2019. Neuro-biological underpinnings of fibromyalgia and centralized pain autonomic dysfunction and fibromyalgia. Clinical and Experimental Rheumatology 37, S129-undefined.
13. Rosner I., Rozenbaum M., Naschitz J.E., Sabo E., Yeshurun D., 2000. Dysautonomia in chronic fatigue syndrome vs. fibromyalgia. The Israel Medical Association journal : IMAJ 2 Suppl.
14. Ruiz N., Lerma C., Martinez L.A., Vargas A., Infante O., Martinez-Lavin M., 2011. Correlational analysis of heart rate variability parameters with fibromyalgia symptoms. Journal of Rheumatology 38, 1207-undefined. https://doi.org/10.3899/jrheum.110506
15. Santiago T.L., Peixoto D., Costa C., Pereira da Silva J.A., 2017. Cross-sectional analysis of the autonomic nervous system (heart rate variability): correlations with psychological dimensions in women with fibromialgia, rheumatoid arthritis and healthy controls. Annals of the Rheumatic Diseases 76, 1381-undefined. https://doi.org/10.1136/annrheumdis-2017-eular.5974
16. Sohn R., Assar T., Kaufhold I., Brenneis M., Braun S., Junker M., Zaucke F., Pongratz G., Jenei-Lanzl Z., 2024b. EARLY- AND LATE-STAGE OSTEOARTHRITIS PATIENTS EXHIBIT AN AUTONOMIC DYSFUNCTION. Osteoarthritis and Cartilage 32, S61-undefined. https://doi.org/10.1016/j.joca.2024.02.093
17. Sohn R., Assar T., Kaufhold I., Brenneis M., Braun S., Junker M., Zaucke F., Pongratz G., Jenei-Lanzl Z., 2024c. AUTONOMIC (DYS)FUNCTION IN LATE-STAGE OSTEOARTHRITIS PATIENTS RECOVERS ONE YEAR AFTER TOTAL KNEE ARTHROPLASTY. Osteoarthritis and Cartilage 32. https://doi.org/10.1016/j.joca.2024.02.156
18. Themelis K., Stocks R., Tano P., Cipinova Z., Shah-Goodwin L., Barritt A., Critchley H., Davies K., Eccles J., 2019. Autonomic and inflammatory changes in FM and ME/CFS and the contribution to signs and Symptoms. Annals of the Rheumatic Diseases 78, 1920-undefined. https://doi.org/10.1136/annrheumdis-2019-eular.1917
19. Treister R., Lodahl M., Lang M., Oaklander A.L., 2015. Development of a comprehensive symptom survey for small-fiber polyneuropathy (SFPN). Annals of Neurology 78. https://doi.org/10.1002/ana.24498
20. Triantafyllias K., Stortz M., DeBlasi M., Leistner C., Weinmann-Menke J., Schwarting A., 2018. Assessment of cardiovascular risk in patients with fibromyalgia by carotidfemoral pulse wave velocity-results of a prospective study. Annals of the Rheumatic Diseases 77. https://doi.org/10.1136/annrheumdis-2018-eular.6145
21. Vincent A., McAllister S., Oh T., Whipple M., 2013. Autonomic symptom profile and fibromyalgia. Journal of Pain 14, S57-undefined. <https://doi.org/10.1016/j.jpain.2013.01.565>

None-relevant outcome

1. Barbic F., Diana A., Casella F., Perego F., Borella M., Dipaola F., Costantino G., Longhi P., Rubin P., Repetti V., Furlan R., 2010. Relationship between sympathetic activity and pain intensity in Fibromyalgia. Clinical Autonomic Research 20. https://doi.org/10.1007/s10286-010-0060-z
2. Boneparth, A., Chen, S., Horton, D.B., Moorthy, L.N., Farquhar, I., Downs, H.M., Lee, H., Oaklander, A.L., 2021. Epidermal Neurite Density in Skin Biopsies From Patients With Juvenile Fibromyalgia. The Journal of rheumatology 48. https://doi.org/10.3899/jrheum.200378
3. Eisenlohr-Moul T.A., Crofford L.J., Howard T.W., Yepes J.F., Carlson C.R., De Leeuw R., 2015. Parasympathetic reactivity in fibromyalgia and temporomandibular disorder: Associations with sleep problems, symptom severity, and functional impairment. Journal of Pain 16. https://doi.org/10.1016/j.jpain.2014.12.005
4. Garcia-Hernandez, A., de la Coba, P., Reyes Del Paso, G.A., 2022. Central sensitisation pain and autonomic deficiencies in fibromyalgia. Clin Exp Rheumatol 40, 1202–1209. https://doi.org/10.55563/clinexprheumatol/n280oi
5. Gavi M.B.R.O., Vassalo D.V., Amaral F.T., Macedo D.C.F., Gava P.L., Dantas E.M., Valim V., 2014. Strengthening exercises improve symptoms and quality of life but do not change autonomic modulation in fibromyalgia: A randomized clinical trial. PLoS ONE 9. https://doi.org/10.1371/journal.pone.0090767
6. Glasgow, A., Stone, T.M., Kingsley, J.D., 2017. Resistance Exercise Training on Disease Impact, Pain Catastrophizing and Autonomic Modulation in Women with Fibromyalgia. International journal of exercise science 10.
7. Hackshaw, K.V., Yao, S., Bao, H., de Lamo Castellvi, S., Aziz, R., Nuguri, S.M., Yu, L., Osuna-Diaz, M.M., Brode, W.M., Sebastian, K.R., Giusti, M.M., Rodriguez-Saona, L., 2023. Metabolic Fingerprinting for the Diagnosis of Clinically Similar Long COVID and Fibromyalgia Using a Portable FT-MIR Spectroscopic Combined with Chemometrics. Biomedicines 11. https://doi.org/10.3390/biomedicines11102704
8. Hassanpour S., Algitami H., Umraw M., Merletti J., Keast B., Stroman P.W., 2024. Investigating Descending Pain Regulation in Fibromyalgia and the Link to Altered Autonomic Regulation by Means of Functional MRI Data. Brain Sciences 14. <https://doi.org/10.3390/brainsci14050450>
9. LLedo Boyer A., Lopez-Roig S., Pastor-Mira M., Terol Cantero M.C., Martin-Aragon M., 2024. Exploring Biopsychosocial Stress Markers in Women With Fibromyalgia. Pain management nursing : official journal of the American Society of Pain Management Nurses 25. https://doi.org/10.1016/j.pmn.2024.05.008
10. Martinez-Lavin M., Hermosillo A.G., Rosas M., Soto M.-E., 1998. Circadian studies of autonomic nervous balance in patients with fibromyalgia: A heart rate variability analysis. Arthritis and Rheumatism 41. https://doi.org/10.1002/1529-0131(199811)41:11<1966::aid-art11>3.3.co;2-f
11. Malysheva O., Baum P., Voitzsch A., Baerwald C.G.O., 2010. Differences in the autonomic reactivity pattern upon various stressors in patients with rheumatoid arthritis. Arthritis and Rheumatism 62. <https://doi.org/10.1002/art.28792>
12. Moreira R.M., Rosario R.C., Boggiss E.A., de Lima R.A., Silva P.A., da Silva K.P., de Farias C.L., dos Santos V.D.Q., da Silva J.R.T., Simoes R.P., Terra A.M.S.V., Santos A.T.S., 2023. Effect of Systemic and Auricular Acupuncture with a 2/100 Hz Frequency and Nogier Frequency in Fibromyalgia: a Randomized Clinical Trial, Pilot Study. JAMS Journal of Acupuncture and Meridian Studies 16. https://doi.org/10.51507/j.jams.2023.16.4.139
13. Schamne J.C., Ressetti J.C., Lima-Silva A.E., Okuno N.M., 2021. Impaired Cardiac Autonomic Control in Women With Fibromyalgia Is Independent of Their Physical Fitness. Journal of Clinical Rheumatology 27. https://doi.org/10.1097/RHU.0000000000001518
14. Shanmugam S., Mathias L., Thakur A., Kumar D., 2016. Effects of intramuscular electrical stimulation using inversely placed electrodes on myofascial pain syndrome in the shoulder - A case series. Korean Journal of Pain 29. <https://doi.org/10.3344/kjp.2016.29.2.136>
15. Wood P.B., Holman A.J., 2009. Treatment of fibromyalgia with pindolol: A 12-week, open-label, fixed dose escalation, observational study. Arthritis and Rheumatism 60. https://doi.org/10.1002/art.25181
16. Zaproudina N., Ming Z., Hanninen O.O.P., 2006. Plantar Infrared Thermography Measurements and Low Back Pain Intensity. Journal of Manipulative and Physiological Therapeutics 29. https://doi.org/10.1016/j.jmpt.2006.01.003

No prevalence estimation

1. Carvalho, H.C., Machado, N.C.S.S., Yáñez-Silva, A., Rocabado, M., Júnior, A.R. de P., Alves, L.P., Ribeiro, W., Lazo-Osório, R.A., 2022. Autonomic nerve regulation in joint hypermobility patients with myofascial trigger points by Musculoskeletal Interfiber Counterirritant Stimulation (MICS). Medical Engineering & Physics 109, 103903. https://doi.org/10.1016/j.medengphy.2022.103903
2. Chalaye, P., Goffaux, P., Bourgault, P., Lafrenaye, S., Devroede, G., Watier, A., Marchand, S., 2012. Comparing Pain Modulation and Autonomic Responses in Fibromyalgia and Irritable Bowel Syndrome Patients. The Clinical Journal of Pain 28, 519. https://doi.org/10.1097/AJP.0b013e31823ae69e
3. Chaves A.C.S., Reis F.J.J., Bandeira P.M., Fernandes O., Arruda Sanchez T., 2021. Autonomic dysregulation and impairments in the recognition of facial emotional expressions in patients with chronic musculoskeletal pain. Scandinavian Journal of Pain 21, 530–538. <https://doi.org/10.1515/sjpain-2020-0132>
4. Cohen H., Neumann L., Alhosshle A., Kotler M., Abu-Shakra M., Buskila D., 2001. Abnormal sympathovagal balance in men with fibromyalgia. Journal of Rheumatology 28.
5. Cohen H., Neumann L., Shore M., Amir M., Cassuto Y., Buskila D., 2000. Autonomic dysfunction in patients with fibromyalgia: Application of power spectral analysis of heart rate variability. Seminars in Arthritis and Rheumatism 29, 217–227. https://doi.org/10.1016/S0049-0172(00)80010-4
6. Contreras-Merino A.M., Davydov D.M., Galvez-Sanchez C.M., Reyes del Paso G.A., 2022. Blunted short-term autonomic cardiovascular reactivity to orthostatic and clinostatic challenges in fibromyalgia as an indicator of the severity of chronic pain. International Journal of Psychophysiology 175, 61–70. https://doi.org/10.1016/j.ijpsycho.2022.03.001
7. Costa A.R., Freire A., Parraca J.A., Silva V., Tomas-Carus P., Villafaina S., 2022. Heart Rate Variability and Salivary Biomarkers Differences between Fibromyalgia and Healthy Participants after an Exercise Fatigue Protocol: An Experimental Study. Diagnostics 12. https://doi.org/10.3390/diagnostics12092220
8. da Cunha Ribeiro, R.P., Roschel, H., Artioli, G.G., Dassouki, T., Perandini, L.A., Calich, A.L., de Sá Pinto, A.L., Lima, F.R., Bonfá, E., Gualano, B., 2011. Cardiac autonomic impairment and chronotropic incompetence in fibromyalgia. Arthritis Res Ther 13, R190. https://doi.org/10.1186/ar3519
9. Di Franco M., Iannuccelli C., Alessandri C., Paradiso M., Riccieri V., Libri F., Valesini G., 2009. Autonomic dysfunction and neuropeptide Y in fibromyalgia. Clinical and experimental rheumatology 27, S75-78.
10. Eccles J.A., Aslanyan D., Harrison N.A., Davies K.A., Critchley H.D., 2017. Dissociative experiences in patients with fibromyalgia are mediated by symptoms of autonomic dysfunction. Journal of Neurology, Neurosurgery and Psychiatry 88. <https://doi.org/10.1136/jnnp-2017-BNPA.44>
11. Hassett A.L., Radvanski D.C., Vaschillo E.G., Vaschillo B., Sigal L.H., Karavidas M.K., Buyske S., Lehrer P.M., 2007. A pilot study of the efficacy of heart rate variability (HRV) biofeedback in patients with fibromyalgia. Applied Psychophysiology Biofeedback 32. https://doi.org/10.1007/s10484-006-9028-0
12. Hazra S., Venkataraman S., Handa G., Yadav S.L., Wadhwa S., Singh U., Kochhar K.P., Deepak K.K., Sarkar K., 2020. A cross-sectional study on central sensitization and autonomic changes in fibromyalgia. Frontiers in Neuroscience 14, 788-undefined. https://doi.org/10.3389/fnins.2020.00788
13. He W., Sheng Z.-M., Wang L., Gaischek I., Litscher G., 2013. Modulation of autonomic nervous system during and after acupuncture treatment of lumbosacral pain in women: A preliminary clinical observational study. Medical Acupuncture 25. https://doi.org/10.1089/acu.2012.0884
14. Kang, J.H., Kim, J.K., Hong, S.H., Lee, C.H., Choi, B.Y., 2016. Heart Rate Variability for Quantification of Autonomic Dysfunction in Fibromyalgia. Annals of rehabilitation medicine 40, 301–9. <https://doi.org/10.5535/arm.2016.40.2.301>
15. Kulshreshtha P., Gupta R., Yadav R.K., Bijlani R.L., Deepak K.K., 2012. A comprehensive study of autonomic dysfunction in the fibromyalgia patients. Clinical Autonomic Research 22, 117–122. https://doi.org/10.1007/s10286-011-0150-6
16. Lodahl, M., Treister, R., Oaklander, A.L., 2018. Specific symptoms may discriminate between fibromyalgia patients with vs without objective test evidence of small-fiber polyneuropathy. Pain reports 3, e633-undefined. <https://doi.org/10.1097/PR9.0000000000000633>
17. Mikkonen J., Kupari S., Tarvainen M., Neblett R., Airaksinen O., Luomajoki H., Leinonen V., 2024. To what degree patient-reported symptoms of central sensitization, kinesiophobia, disability, sleep, and life quality associated with 24-h heart rate variability and actigraphy measurements? Pain Practice 24. <https://doi.org/10.1111/papr.13331>
18. Morikawa Y., Takamoto K., Nishimaru H., Taguchi T., Urakawa S., Sakai S., Ono T., Nishijo H., 2017. Compression at myofascial trigger point on chronic neck pain provides pain relief through the prefrontal cortex and autonomic nervous system: A pilot study. Frontiers in Neuroscience 11. <https://doi.org/10.3389/fnins.2017.00186>
19. Shankar N., Thakur M., Tandon O.P., Saxena A.K., Arora S., Bhattacharya N., 2011. Autonomic status and pain profile in patients of chronic low back pain and following electro acupuncture therapy: A randomized control trial. Indian Journal of Physiology and Pharmacology 55, 25–36.
20. On A.Y., Tanigor G., Baydar D.A., 2022. Relationships of autonomic dysfunction with disease severity and neuropathic pain features in fibromyalgia: Is it really a sympathetically maintained neuropathic pain? Korean Journal of Pain 35, 327–335. <https://doi.org/10.3344/kjp.2022.35.3.327>
21. Pardo J.V., Larson R.C., Spencer R.J., Lee J.T., Pasley J.D., Torkelson C.J., Larson A.A., 2019. Exposure to Cold Unmasks Potential Biomarkers of Fibromyalgia Syndrome Reflecting Insufficient Sympathetic Responses to Stress. Clinical Journal of Pain 35. https://doi.org/10.1097/AJP.0000000000000695
22. Prados, G., Miró, E., Martínez, M.P., Sánchez, A.I., Pichot, V., Medina-Casado, M., Chouchou, F., 2022. Effect of Cognitive-Behavioral Therapy on Nocturnal Autonomic Activity in Patients with Fibromyalgia: A Preliminary Study. Brain Sci 12, 947. https://doi.org/10.3390/brainsci12070947
23. Puri, B.K. and Lee, G.S., 2023. The principal components of autonomic dysfunction in fibromyalgia assessed by the refined and abbreviated Composite Autonomic Symptom Score. Reviews on Recent Clinical Trials, 18(2), pp.140–145. https://doi.org/10.2174/1574887118666230315120413
24. Ramirez M., Guerra-Juarez A., Miyake D.-Y., Sebastian-Arellano C., Estrada-Mata A.-G., Gonzalez-Moyotl N.-J., Rodriguez-Aguayo A.-M., Martinez-Lavin M., Martinez-Martinez L.-A., 2021. Correlation between Corneal Nerve Density and Symptoms of Small Fiber Neuropathy in Patients with Fibromyalgia: The Confounding Role of Severe Anxiety or Depression. Journal of Clinical Rheumatology 27. <https://doi.org/10.1097/RHU.0000000000001592>
25. Reis M.S., Durigan J.L.Q., Arena R., Rossi B.R.O., Mendes R.G., Borghi-Silva A., 2014. Effects of posteroanterior thoracic mobilization on heart rate variability and pain in women with fibromyalgia. Rehabilitation Research and Practice 2014. https://doi.org/10.1155/2014/898763
26. Reyes Del Paso G.A., Contreras-Merino A.M., Duschek S., 2022. The Role of Depressive Disorders in Autonomic Cardiovascular Dysregulation in Fibromyalgia. Psychosomatic Medicine 84, 793–802. https://doi.org/10.1097/PSY.0000000000001097
27. Reyes del Paso G.A., de la Coba P., 2020. Reduced activity, reactivity and functionality of the sympathetic nervous system in fibromyalgia: An electrodermal study. PLoS ONE 15. <https://doi.org/10.1371/journal.pone.0241154>
28. Roy A., Singh S.K., Tiwari A.K., 2017. Evaluation of autonomic responses in male patients suffering with chronic pain using Valsalva maneuver and tilt table test. Research Journal of Pharmaceutical, Biological and Chemical Sciences 8, 1382–1389.
29. Singh S.K., Roy A., 2017. Assessment of heart rate variability in the patients suffering with chronic pain of musculoskeletal origin. National Journal of Physiology, Pharmacy and Pharmacology 7, 712–718. https://doi.org/10.5455/njppp.2017.7.0204803032017
30. Sochodolak, R.C., Schamne, J.C., Ressetti, J.C., Costa, B.M., Antunes, E.L., Okuno, N.M., 2022. A comparative study of heart rate variability and physical fitness in women with moderate and severe fibromyalgia. Journal of exercise rehabilitation 18, 133–140. https://doi.org/10.12965/jer.2244070.035
31. Sohn R., Assar T., Kaufhold I., Brenneis M., Braun S., Junker M., Zaucke F., Pongratz G., Jenei-Lanzl Z., 2024a. Osteoarthritis patients exhibit an autonomic dysfunction with indirect sympathetic dominance. Journal of Translational Medicine 22, 467-undefined. <https://doi.org/10.1186/s12967-024-05258-9>
32. Unlu E., Ulas U.H., Gurcay E., Tuncay R., Berber S., Cakci A., Odabasi Z., 2006. Genital sympathetic skin responses in fibromyalgia syndrome. Rheumatology International 26. <https://doi.org/10.1007/s00296-006-0131-6>
33. Yeater, T.D., Clark, D.J., Hoyos, L., Valdes-Hernandez, P.A., Peraza, J.A., Allen, K.D., Cruz-Almeida, Y., 2021. Chronic Pain is Associated With Reduced Sympathetic Nervous System Reactivity During Simple and Complex Walking Tasks: Potential Cerebral Mechanisms. Chronic stress (Thousand Oaks, Calif.) 5, 24705470211030273-undefined. <https://doi.org/10.1177/24705470211030273>
34. Zetterman T., Markkula R., Miettinen T., Kalso E., 2023. Heart rate variability responses to cognitive stress in fibromyalgia are characterised by inadequate autonomous system stress responses: a clinical trial. Scientific reports 13, 700-undefined. https://doi.org/10.1038/s41598-023-27581-9

Potential sample overlap

1. Naschitz J.E., Rosner I., Rozenbaum M., Naschitz S., Musafia-Priselac R., Shaviv N., Fields M., Isseroff H., Zuckerman E., Yeshurun D., Sabo E., 2003. The head-up tilt test with haemodynamic instability score in diagnosing chronic fatigue syndrome. QJM: An International Journal of Medicine 96, 133–142. https://doi.org/10.1093/qjmed/hcg018

## Supplementary Table 3. Adapted Newcastle - Ottawa Quality Assessment Scale

Note: A study can be awarded a maximum of one star for each numbered item within the Selection and comparability categories. A maximum of two stars can be given for **Comprehensiveness of the used method**

**Selection**

**1) Is the definition of the musculoskeletal condition adequate?**

a) Yes, clinically approved. *

b) No, Self-diagnosed – not lab or clinically confirmed

**2) Representativeness of the cases**

a) Consecutive or obviously representative series of cases *

b) Potential for selection biases or not stated

**3) Comparison with controls**

a) yes *

b) no

**Comparability**

**1) Comparability of cases and controls based on the design or analysis**

a) Controls are comparable to cases in terms of setting, demographics, and other confounding variables. *

b) Controls are recruited from a different site or are not adequately matched to cases in terms of key variables.

c) No control group included.

**Outcome**

1) **Were all study participants tested for dysautonomia?**

a) Yes. *

b) No. 

**2) Validity of the method used to assess dysautonomia**

a) Validated objective or subjective method *

b) Non-validated method

3) **Comprehensiveness of the method used to assess dysautonomia**

a) Assessment included a comprehensive battery of validated autonomic tests covering multiple domains *

b) Assessment used only a single validated test or a limited subset without full domain coverage

Risk of Bias Classification:

6–7 stars Low Risk

4–5 stars Moderate Risk

0–3 stars High Risk

## Supplementary Table 4. Risk of Bias Assessment

Risk of bias ratings for included studies using the adapted Newcastle–Ottawa Quality Assessment Scale (maximum score: 7 stars).

| **Study** | Diagnosis Adequacy | Sample Representativeness | Comparison with controls | Comparability | Were all study participants tested for dysautonomia? | Validity of Dysautonomia Assessment | Comprehensiveness of Assessment | **Total Score** |
| --- | --- | --- | --- | --- | --- | --- | --- | --- |
| Visuri, et al. 1992 | Diagnosis of FM confirmed by clinicians, using published criteria | n=17 FM  n=20 controls | Yes | Controls similar mean age and same sex | The response rate is not mentioned or calculable | Active standing test. Validated questionnaire of dystonic symptoms. | Yes | 5 |
|  | * |  | * | * |  | * | * |  |
| Tang, et al. 2004 | FM assessed using ACR criteria | n=76 with SLE  (18 FM) | No | N/A  No health controls | 27.6% of participants withdrew before study completion - the withdrawal reasons were outlined and are unlikely to introduce bias. | NMH measured using validated tool (tilt-table test). | single validated test or a limited subset | 4 |
|  | * |  |  | N/A | * | * | * |  |
| Furlan, et al. 2005 | FM diagnosis confirmed using ACR criteria and inclusion criteria | n= 16 Consecutive patients from a hospital-based rheumatology clinic. | Yes | Study controls for age and sex through matching. | Yes | Used structured HRV and autonomic testing protocols. | Yes | 7 |
|  | * | * | * | * | * | * | * |  |
| Naschitz, et al. 2006 | FM used ACR criteria | n=70 FM  n=50 controls | yes | Controls younger | Analysis includes data from all participants, no mention of dropouts, missing data, or exclusions. | Validated tool (tilt-table test) | single validated test or a limited subset | 5 |
|  | * |  | * |  | * | * | * |  |
| Seidel, et al. 2007 | Fibromyalgia diagnosis confirmed using ACR criteria and inclusion criteria clearly outlined. | n=72 patients from a rehabilitation center.  Sampling strategy not stated. All females. Few descriptive statistics are provided to confirm representative. | Yes | Study controls for age and sex through matching. | Yes | Used ISAX—a validated tool for HRV and autonomic function measurements. | No | 5 |
|  | * |  | * | * | * | * |  |  |
| Stojanovich, et al. 2007 | Diagnosis of chronic musculoskeletal autoimmune conditions (SLE, RA, etc.) made using ACR criteria—clear and validated. | n= 125 patients from a university medical center and 35 controls. Sampling strategy is not stated, but descriptive statistics confirm representative. | Yes | Control group formed to approximately frequency match the mean age. Sex ratio differed substantially. No individual-matching or statistical control for major confounders such as age, BMI, or comorbidities. | Yes | autonomic dysfunction assessed using gold-standard cardiovascular reflex tests Ewing’s battery | Yes | 6 |
|  | * | * | * |  | * | * | * |  |
| Solano, et al. 2009 | FM and RA used ACR criteria | n=30 FM  n=30 RA  n=30 controls | Yes | Controls younger | All patients completed the questionnaires. No indication of dropouts or incomplete participation | Validated questionnaire (COMPASS) | Yes | 5 |
|  | ***** |  | * |  | * | * | * |  |
| El-Sawy, et al. 2012 | FM used ACR criteria | n=25 FM  n=15 controls | Yes | Controls similar mean age and sex ratio | The response rate is not mentioned or calculable | Objective measures: tilt table test and sympathetic skin response (SSR) | No | 4 |
|  | * |  | * | * |  | * |  |  |
| Oaklander, et al 2013 | FM used ACR criteria | n=57 FM | Yes | Controls similar mean age and sex ratio. | 64% response rate for FM subjects | autonomic-function testing (AFT) | Yes | 5 |
|  | * |  | * | * |  | * | * |  |
| De Wandele, et al. 2014a | Diagnosis of EDS-HT confirmed by clinicians | n=74 EDS-HT  n=35 controls | Yes | Controls similar mean age and same sex | The response rate is not mentioned or calculable | Used Composite Autonomic Scoring Scale (CASS), a validated tool | Yes | 5 |
|  | * |  | * | * |  | * | * |  |
| De Wandele, et al. 2014b | Diagnosis of EDS-HT confirmed by clinicians | n=80 EDS-HT | No | N/A  No healthy controls with prevalence of dysautonomia reported | 99% response rate | Used Composite Autonomic Scoring Scale (CASS), validated tool | Yes | 4 |
|  | * |  |  | N/A | * | * | * |  |
| Vincent, et al. 2016 | FM diagnosis documented by a health care provider in medical records | n=30 FM  n=30 controls | Yes | Controls younger, with an unknown % recruited from outside the cohort | Incomplete DXA measures, but all other % out of n=30 | CASS and COMPASS31 | Yes | 5 |
|  | * |  | * |  | * | * | * |  |
| Lee, et al. 2018 | FM used ACR criteria | n=35 FM  n=25 controls | Yes | Controls said to be similar mean age (data not presented). Controls same sex | The response rate is not mentioned or calculable | Ewing tests Standard validated autonomic function tests | Yes | 5 |
|  | * |  | * | * |  | * | * |  |
| Song, et al. 2020 | Diagnosis of EDS and subtypes confirmed by clinicians | n=98 EDS | No | N/A  No healthy controls | Analysis includes data from all participants, no mention of dropouts, missing data, or exclusions. | Presence of autonomic dysfunction confirmed by clinicians | Yes | 4 |
|  | * |  |  | N/A | * | * | * |  |
| Singh, et al. 2021 | FM used ACR criteria | n=30 FM  n=30 controls | Yes | Matched for age and gender | 91% completion rate | Used Ewing’s battery of validated tests | Yes | 6 |
|  | * |  | * | * | * | * | * |  |
| Mucci, et al. 2022 | Diagnosis of FM confirmed by clinicians | n=277 FM  n=80 controls | Yes | Controls similar gender, but younger | FM response rate not stated.  Controls response rate 41% | Validated Dizziness Handicap Inventory (DHI) and Situational Vertigo Ques-  tionnaire (SVQ) | No | 4 |
|  | * | * | * |  |  | * |  |  |
| Kulshreshtha, et al. 2022 | Diagnosis of FM confirmed by clinicians | n=42 FM |  | N/A  No healthy controls with prevalence of dysautonomia reported | The response rate is not mentioned or calculable | Used Ewing’s battery of validated tests | Yes | 3 |
|  | * |  |  | N/A |  | * | * |  |

Risk of Bias Classification:

6–7 stars Low Risk = 3 studies

4–5 stars Moderate Risk = 13 studies

0–3 stars High Risk = 1 studies

## Supplementary Figure 1. PRISMA flowchart demonstrates the article screening process.

**Identification of studies via other methods**

**Identification of studies via databases and registers**

Records identified from:

Citation searching (n=2)

Record removed because of duplication

(n=357)

Records identified through database searching

(n=1093)

**Identification**

Record screened for title/ abstract

(n=736)

Reports excluded:

Conference abstracts (n=29)

Duplication: (n=2)

Not written in English (n=1)

Review article (n=1)

Editorial (n=1)

Not relevant population (n=1)

Full text not available (n=3)

Not relevant (n=1)

Reports sought for retrieval

(n =2)

Reports sought for retrieval

(n=149)

**Screening**

Reports excluded:

Editorial (=3)

Conference abstracts (n=21)

Targeted population (n = 11)

Review article (n=6)

Non-relevent outcome (n=16)

No prevalence estimate (n=34)

No validated tool (n=2)

Potential sample overlap (n=1)

Reports excluded:

Non-relevent outcome (n=1)

Reports assessed for eligibility

(n = 1)

Reports assessed for eligibility

(full text screening)

(n=110)

Studies included in the review

(n = 17)

## Forst plots


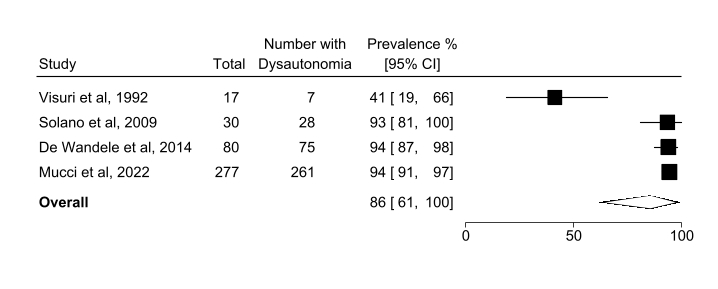
Supplementary Figure 2. Forest plot shows overall pooled prevalence estimates of Dysautonomia across various musculoskeletal conditions using ONLY subjective methods

###
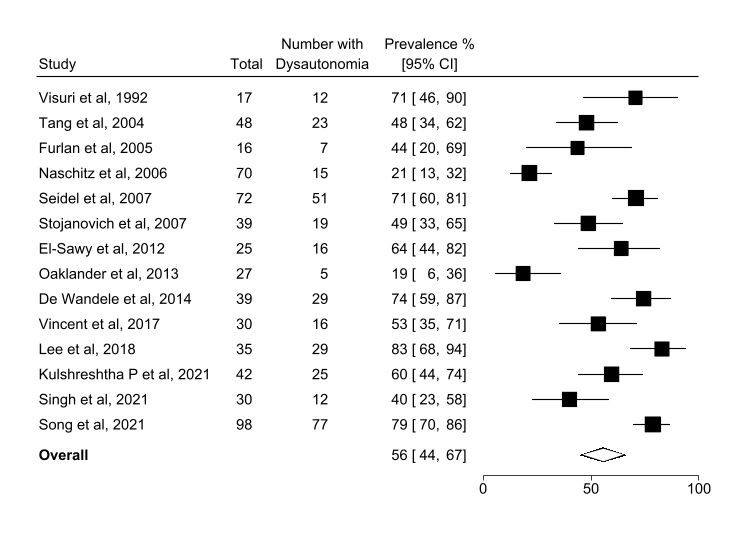
Supplementary Figure 3. Forest plot shows overall pooled prevalence estimates of Dysautonomia across various musculoskeletal conditions using ONLY objective methods


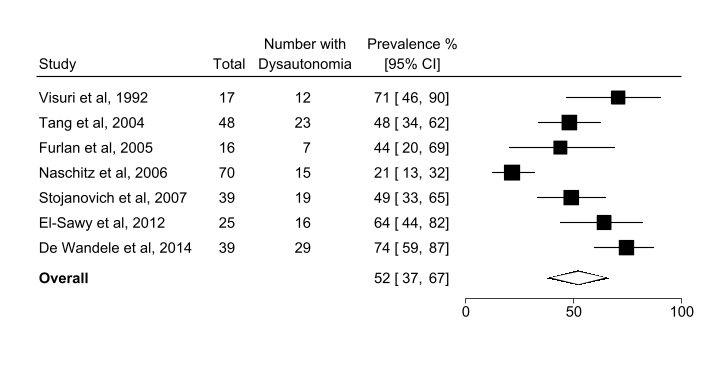
Supplementary Figure 4. Forest plot shows overall pooled prevalence estimates of Dysautonomia in all included musculoskeletal conditions using orthostatic intolerance domain.

### Supplementary Figure 5***.*** Forest plot shows overall pooled prevalence estimates of Dysautonomia in all included musculoskeletal conditions using groups of autonomic tests, and only report a single cardiac-related outcome (cardiac autonomic dysfunction (CAD) or cardiac autonomic neuropathy (CAN)


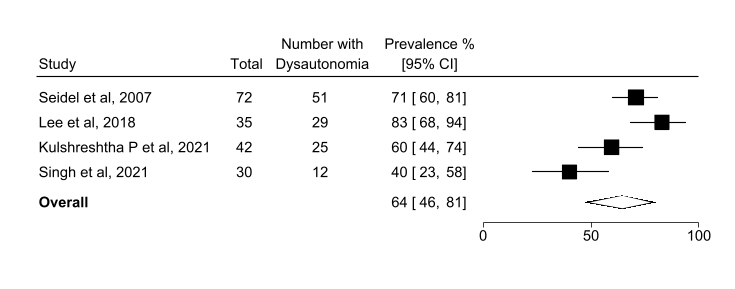


###
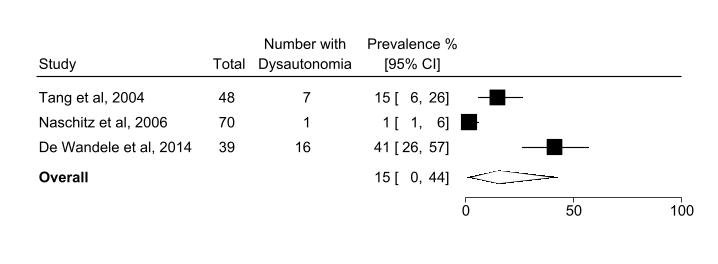
Supplementary Figure 6***.*** Forest plot shows overall pooled prevalence estimates of POTS in all included musculoskeletal conditions using Tilt table test


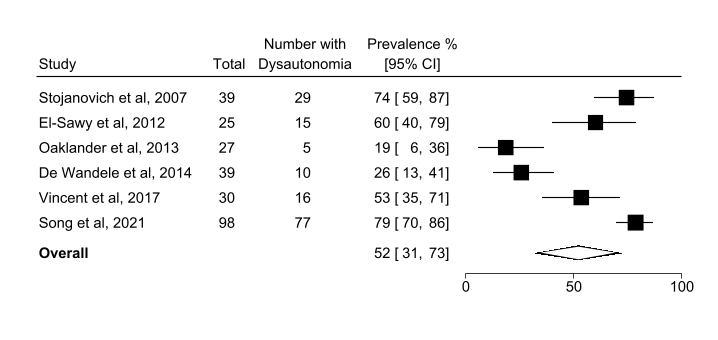
Supplementary Figure 7. Forest plot shows overall pooled prevalence estimates of Dysautonomia in all included musculoskeletal conditions using mixed clinical and subclinical testing approaches


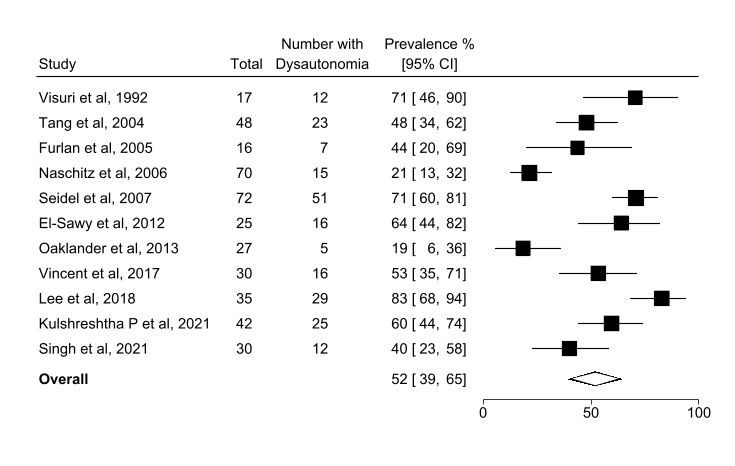
Supplementary Figure 8. Forest plot shows the overall pooled prevalence of dysautonomia in fibromyalgia, based on validated objective assessment methods.


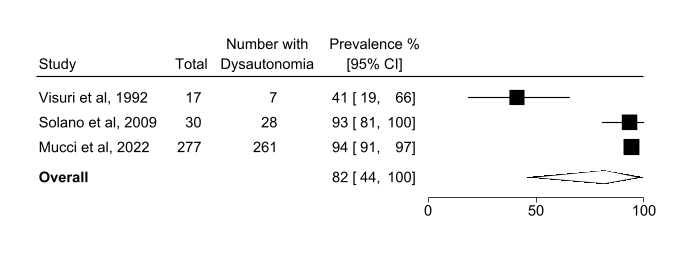
Supplementary Figure 9. Forest plot shows the overall pooled prevalence of dysautonomia in fibromyalgia, using self-report methods.


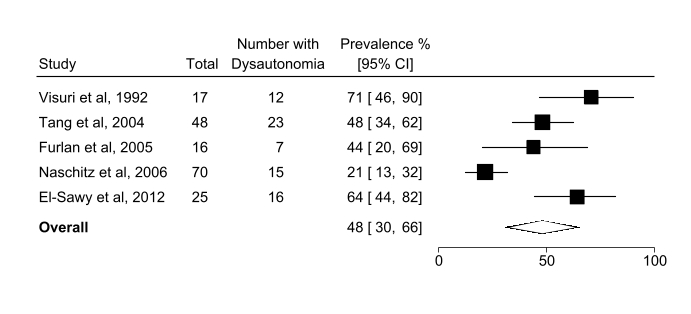
Supplementary Figure 10. Forest plot shows overall pooled prevalence estimates of Dysautonomia in fibromyalgia using orthostatic intolerance domain.

*Supplementary Figure 11. Forest plot shows the overall pooled prevalence of dysautonomia in Ehlers-Danlos syndrome, based on validated objective and subjective assessment methods.*

**
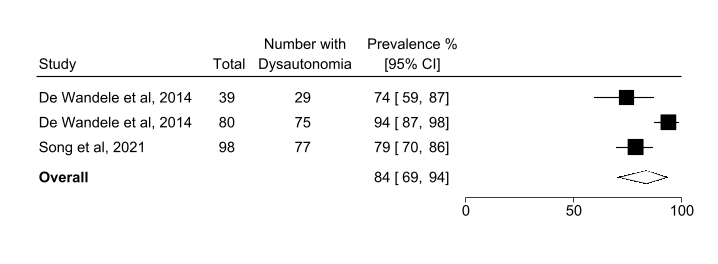
**

## Funnel plots

Supplementary Figure 12. Funnel plot of dysautonomia prevalence estimates across all included studies and assessment methods.


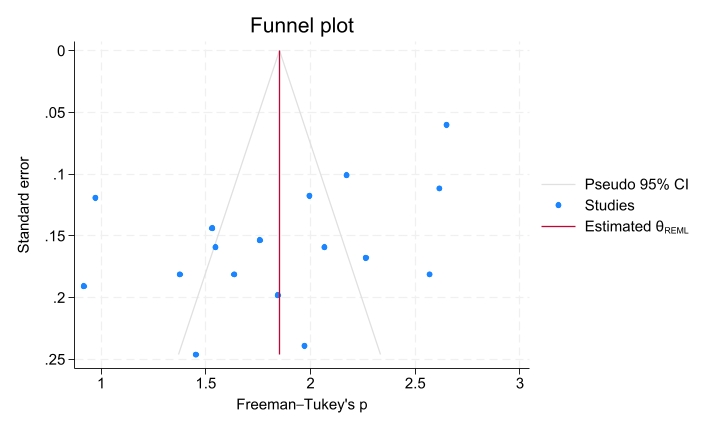


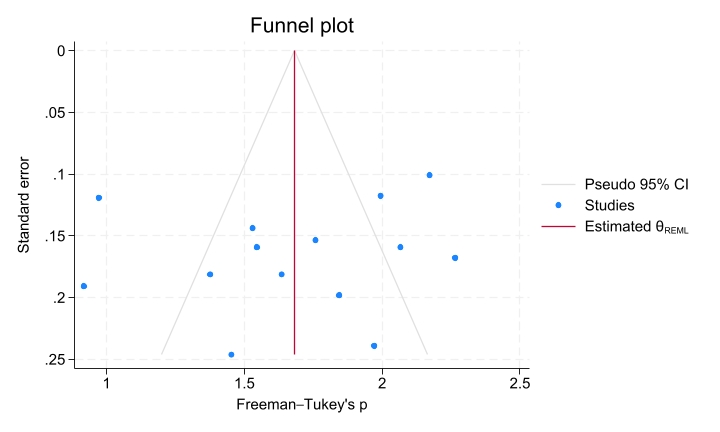
Supplementary Figure 13. Funnel plot of dysautonomia prevalence estimates across all included studies using objective assessment methods


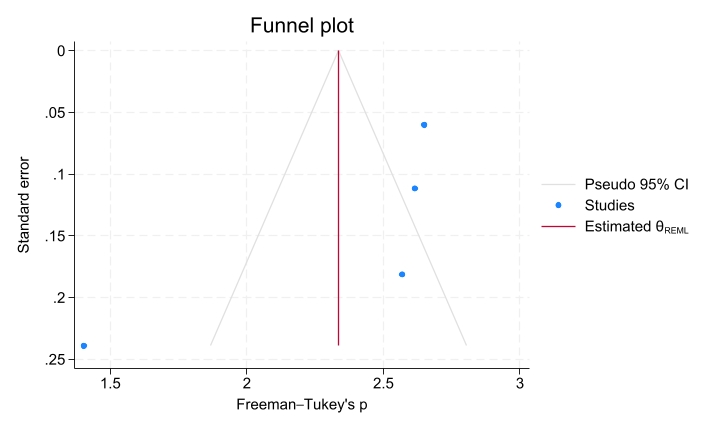
Supplementary Figure14. Funnel plot of dysautonomia prevalence estimates across all included studies using subjective assessment methods

Supplementary Figure 15. Funnel plot of dysautonomia prevalence estimates across all included studies based on orthostatic domain assessment

**
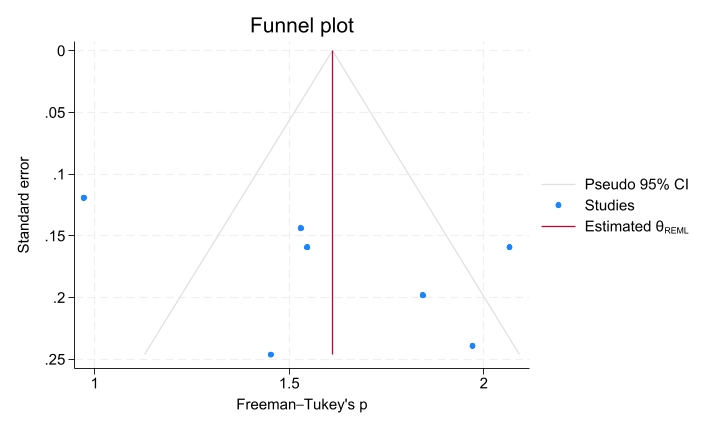
**

Supplementary Figure 16. Funnel plot of dysautonomia prevalence estimates across all included studies based on mixed clinical and subclinical testing approaches.


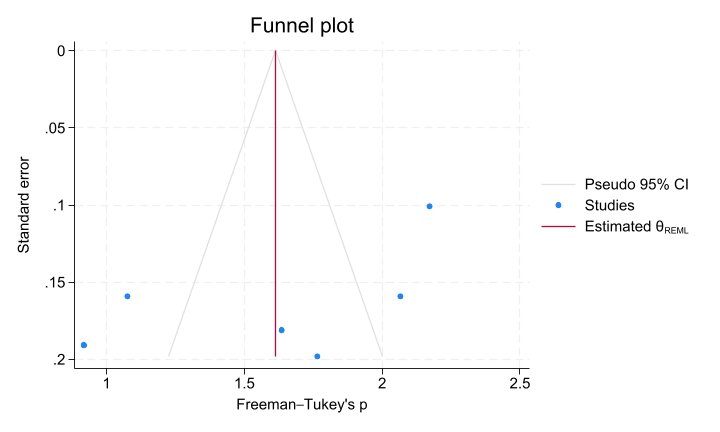


Supplementary Figure 17. Funnel plot of dysautonomia prevalence estimates in fibromyalgia using various assessment methods.


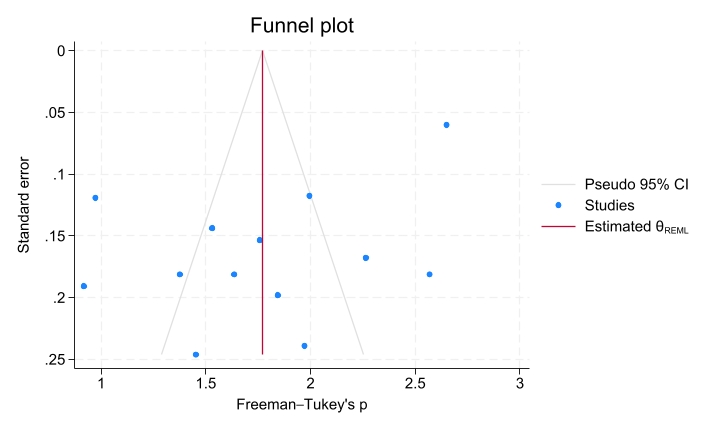


### Supplementary Figure 18***.*** Funnel plot of dysautonomia prevalence estimates in fibromyalgia using objective assessment methods


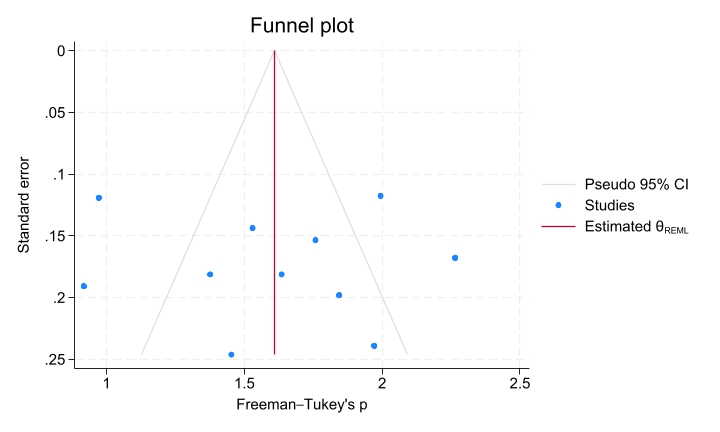


### Supplementary Figure 19. Funnel plot of dysautonomia prevalence estimates in fibromyalgia based on orthostatic domain assessment


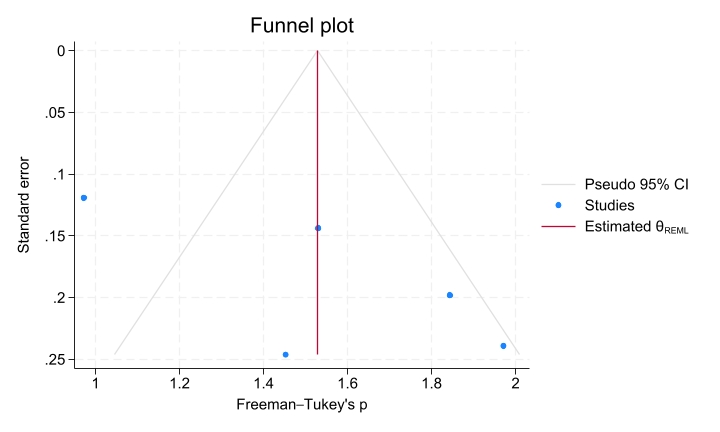

Supplement: rkag034_Supplementary_Data [file rkag034_supplementary_data.zip › RHEUMAP-2025-196.R2._ Supplementary Materials.docx]
